# Quantitative determination of in-plane optical anisotropy by surface plasmon resonance holographic microscopy

**DOI:** 10.1038/s41377-026-02207-7

**Published:** 2026-03-06

**Authors:** Jiwei Zhang, Wenrui Li, Jiahao Li, Yujie Zhang, Xiaoqing Chen, Xiangyuan Luo, Siqing Dai, Xuetao Gan, Jianlin Zhao

**Affiliations:** 1 Key Laboratory of Light Field Manipulation and Information Acquisition, Ministry of Industry and Information Technology, and Shaanxi Key Laboratory of Optical Information Technology, School of Physical Science and Technology, Northwestern Polytechnical University, Xi’an, China; 2 School of Artificial Intelligence, Optics and Electronics (iOPEN), Northwestern Polytechnical University, Xi’an, China

**Keywords:** Imaging and sensing, Interference microscopy

## Abstract

Quantitative determination of in-plane optical anisotropy is essential in finding or designing anisotropic low-dimensional materials and investigating their physical properties. Current determination methods are mostly qualitative or using empirical equations for quantitative calculation. A common weakness of these methods is utilizing light-matter interactions between far-field light and material samples which relies on long interaction distance. However, the thin thickness of low-dimensional material, especially atomic-layer sample, induces an exceeding short light-matter interaction distance and results in low signal-to-noise ratio as well as inaccurate measurement result. In this paper, we propose a novel determination method for in-plane optical anisotropy called azimuthal scanning excitation surface plasmon resonance holographic microscopy. This method utilizes near-field light-matter interactions between material samples and surface plasmon waves oscillating along various in-plane directions. The sample complex refractive indices along all of the in-plane directions can be quantitatively retrieved and thus the magnitude of in-plane optical anisotropy, including birefringence and dichroism, is determined. This method detects the reflection phase shift in surface plasmon resonance regardless of the sample thickness and thus is applicable to ultrathin samples down to atomic-layer. As a demonstration example, monolayer, bilayer and multilayer ReS_2_ samples have been used to verify the validity of the proposed method, and we find that the magnitude of in-plane optical anisotropy increases with the decrease of sample thickness. This work provides a precise determination method for in-plane optical anisotropy of thin film samples with various thickness and gives a guidance in finding new anisotropic low-dimensional materials and engineering new polarized nanodevices.

## Abstract (web summary)

Utilizing near-field light-matter interactions between material samples and surface plasmon waves oscillating along various in-plane directions, the in-plane optical anisotropy of ultrathin samples down to atomic-layer can be precisely determined.

## Graphical abstract

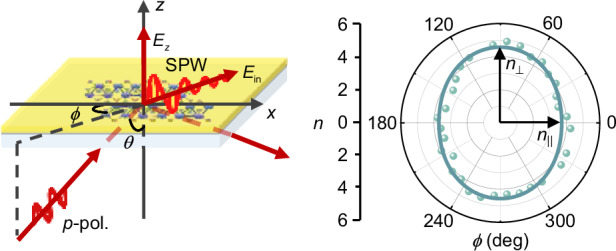

## Introduction

Optical anisotropy is reflected in different optical parameters such as refractive index (RI) and absorption coefficient, when light propagates through materials along different directions. This property exists in in-plane directions, i.e., in-plane optical anisotropy for low-dimensional materials like two-dimensional (2D) materials. In 2014, black phosphorus was first discovered as an in-plane anisotropic 2D material originating from its low-symmetry crystal structure^1^. In recent years, the in-plane optical anisotropy of 2D materials has been widely applied to fabricate anisotropic nanodevices, such as optical waveplates^2,3^, wearable electronics^4^, and polarization-sensitive reflectors^5^, photodetectors^6–9^, perfect absorbers^10^, etc. Attempts to enhance the anisotropic light-matter interactions have pushed researchers to find or artificially design 2D materials with large in-plane anisotropy in a broad spectral range^11–19^. To utilize the in-plane optical anisotropy more effectively, quantitative and precise measurement of the anisotropy is prerequisite.

Since the in-plane optical anisotropy of 2D materials stems from the crystal structures, one straightforward approach to study this property is determining the orientation of crystallographic principal axes. Scanning transmission electron microscope is able to visualize the molecule arrangement and was used to probe the in-plane optical anisotropy of layered ReS_2_^20–22^ and black arsenic^23^. Using the interactions between polarized light and 2D materials, the in-plane optical anisotropy can be determined by detecting the Raman scattering^24–27^, light reflectance and absorption^22,28–33^, photoluminescence (PL)^24,31,34,35^, photothermal^36^, and second harmonic generation (SHG) signals^37–40^. If the polarization state of light can be freely controlled, like polarized Raman spectrometry^21–23,41,42^, the sample will be free of being rotated during the signal detection. Near-field detection approaches have also been used such as nanoimaging of waveguide modes in 2D material crystals with the scanning near-field optical microscope^13,14,43,44^.

Although the above methods are effective, they are only qualitative for probing in-plane optical anisotropy by identifying the crystallographic axis orientation. On the other hand, the anisotropic materials have birefringence and dichroism because of the differences in real and imaginary part of material complex RIs, respectively. Therefore, measuring the in-plane complex RIs of 2D materials becomes a reliable approach to quantitatively analyze the in-plane optical anisotropy. As a commercial instrument, spectrometric ellipsometer has been widely used to measure the birefringence and dichroism of thin films. However, this method is mostly applicable in film samples with micrometer-scale thickness^14,16,32,45,46^ but fails for mono- or few-layer 2D materials. Alternatively, angle-resolved polarized optical contrast spectroscopy has been used to measure the optical contrast of 4 to 110 nm-thick TiS_3_ nanosheets^47^ and 1 to 3-layer ReS_2_ on SiO_2_/Si substrates^48^. Then, a Fresnel-law-based model and empirical equation were built to retrieve the sample complex RIs along different in-plane directions, respectively. These traditional methods use the light-matter interactions between far-field polarized light and 2D materials, the longer the interaction distance, the higher the detected signal-to-noise ratio and measurement accuracy. However, the thin thickness of low-dimensional material, especially for atomic-layer sample always induces exceeding short light-matter interaction distance.

To this end, we propose to put the thin 2D material in optical near field and utilize the light-matter interaction between non-radiant electromagnetic field and the sample. Specifically, we propose an azimuthal scanning excitation surface plasmon resonance (SPR) holographic microscopy (ASE-SPRHM) which generates near-field surface plasmon waves (SPWs) oscillating along various in-plane directions and interacting with the sample to be measured. Based on the dependence of SPR reflection coefficient on the sample physical parameters, complex RIs at all of the in-plane directions can be quantitatively retrieved by measuring the complex amplitude of reflection waves in SPR using digital holography. The experiment results for ReS_2_ samples with various thicknesses verify the validity of this method.

## Results

### Visualizing in-plane optical anisotropy using ASE-SPRHM

**Fig. 1:**
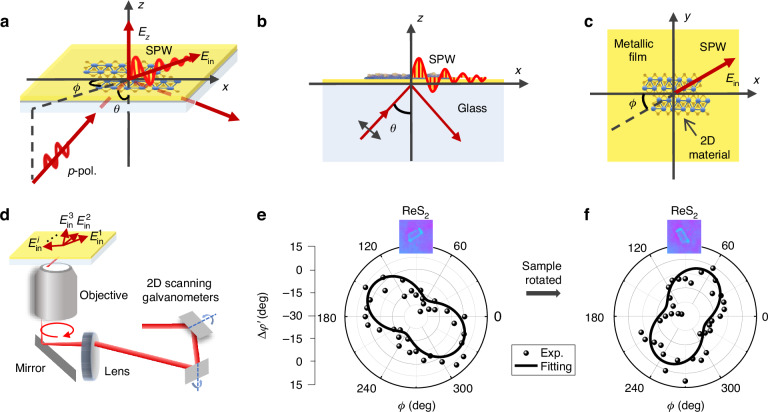
Principle and experiment result of visualizing in-plane optical anisotropy using ASE-SPRHM. **a** Kretschmann configuration of four-layer SPR model. **b** Front-view (*x*-*z* plane) of the configuration when *ϕ*=0. **c** Top-view (*x*-*y* plane) of the configuration. **d** Schematic of azimuthal scanning excitation of SPR. SPWs propagating in various directions produce electric fields oscillating along varying in-plane directions (${E}_{{\rm{in}}}^{1}$, ${E}_{{\rm{in}}}^{2}$, ${E}_{{\rm{in}}}^{3}$,…${E}_{{\rm{in}}}^{i}$). **e** Measured reflection phase shift difference Δ*φ'* of the layered ReS_2_ sample area versus in-plane angle *ϕ* when the 4^th^ dielectric above the sample is water and air. The dots represent the experiment results and closed line the fitting results. **f** Result when the sample is rotated by an angle

In this work, Kretschmann configuration consisting of a glass substrate coated with a thin metallic film is used to excite SPR. When the incident angle of *p*-polarization light beam *θ* is larger than the critical angle and the wavevector matches with that of propagating SPW in optical near field, SPR is excited and the energy of incident light is coupled into SPW (Fig. 1a–c). The reflection coefficient *r* of reflected beam including reflectivity *R* and phase shift *φ* is sensitively modulated by the wavelength *λ* and incident angle *θ* of excitation light, and the parameters of Kretschmann configuration such as the RI of glass substrate *n*_1_, the thickness and permittivity of metallic film (*d*_2_, *ε*_2_) and 3^rd^ dielectric layer (*d*_3_, *ε*_3_) above the metallic film. Since the 3^rd^ dielectric layer in this work is a thin film sample which is totally located in optical near field, the reflection coefficient *r* is also affected by the RI of 4^th^ dielectric above the film sample *n*_4_. The quantitative relationship is determined by the Fresnel formulae (see the details in Supplementary Information S.1). The Kretschmann configuration consisting of glass substrate-metallic film-thin film sample-4^th^ dielectric in this work is called four-layer SPR model. By detecting the reflection coefficient *r*, complex RIs ${\tilde{n}}_{3}(={n}_{3}+{\rm{i}}{k}_{3})$ and thickness *d*_3_ of the thin film sample can be retrieved. SPRHM uses digital holographic microscopy (DHM) to record the hologram which carries the information of complex amplitude of reflection beam in SPR and the SPR intensity and phase images can be numerically reconstructed simultaneously. The SPR images can be further used to retrieve the spatial distribution information of dielectric layer samples^49–60^. The Kretschmann configuration of SPR can be implemented on both the prism and objective structures. In this work, an oil-immersion objective with large numerical aperture is used to capture the high-resolution and distortion-free SPR images (Fig. 1d)^61^.

If the thin film sample has in-plane optical anisotropy, ${\tilde{n}}_{3}$ varies along different in-plane directions. In order to determine the in-plane optical anisotropy, the sample should interact with the SPWs oscillating along different in-plane directions. Apart from the strong longitudinal electric field *E*_*z*_ oscillating along the out-of-plane direction, the SPW also has an in-plane electric field *E*_in_ which oscillates along the propagating direction of SPW (Fig. 1a). By changing the azimuthal angle of SPR excitation light using a pair of 2D scanning galvanometers and adjusting the light polarization state with a vortex half wave plate, SPW will propagate at arbitrary in-plane angle *ϕ* and the corresponding in-plane electric fields oscillating along varying directions (${E}_{{\rm{in}}}^{1}$, ${E}_{{\rm{in}}}^{2}$, ${E}_{{\rm{in}}}^{3}$,…${E}_{{\rm{in}}}^{i}$) will be generated (Fig. 1d). We call this excitation approach of SPR as azimuthal scanning excitation (ASE)^62^. Incorporating this approach with DHM, we employ ASE-SPRHM to determine the in-plane optical anisotropy of thin film samples.

Here, a representative sample, *i.e*., layered ReS_2_ which has strong in-plane optical anisotropy^63^ is used to verify the validity of the proposed method. Using the designed experimental setup of ASE-SPRHM (see the details in Supplementary Information S.2), a series of 36 holograms was recorded when the in-plane angle *ϕ* was scanned around a circle at an interval of 10 degrees on condition that the sample area was under the SPR condition. The SPR intensity and phase images were numerically reconstructed with the angular spectrum method of DHM^64,65^. To remove the background noise in SPR phase image, the reference holograms were recorded by putting a drop of water above the film sample and breaking the SPR condition. By dividing the reconstructed complex amplitudes of object holograms by those of reference holograms, SPR phase difference distributions at multiple in-plane angles were obtained. Then the averaged phase difference values of the sample area Δ*φ'* were calculated and plotted with the in-plane angle *ϕ* in a polar coordinate system, as shown in Fig. 1e. The dots represent the experiment results and closed line the fitting results. The specific fitting equation is Δ*φ'* *=* A sin(2*ϕ* *+* *ϕ*_0_) + *b*, where *A* **=** (Δ*φ'*_max_- Δ*φ'*_min_)/2 is the variation amplitude of Δ*φ'*, *ϕ*_0_ is the initial phase of sinusoidal function, which is determined by the in-plane angle of Δ*φ'*_max_, and *b* is the offset of sinusoidal function. The plot result reveals an elliptical shape between Δ*φ'* and *ϕ*, demonstrating the in-plane optical anisotropy of film sample. If the sample is rotated by an angle, the long axis of elliptical shape plot is rotated accordingly (Fig. 1f), which further confirms that the elliptical shape plot results from the in-plane optical anisotropy of sample. Furthermore, a control experiment was conducted on a layered graphene sample which is in-plane isotropic. The corresponding plot between Δ*φ'* and *ϕ* appears as a circular shape in the polar coordinate system (Supplementary Information S.3).

### Quantitative retrieval of complex RIs and thickness of thin films

The elliptical shape plots between reflection phase shift difference Δ*φ'* of the ReS_2_ sample area and in-plane angle *ϕ* in Fig. 1e, f directly show the in-plane optical anisotropy in a qualitative way. To quantitatively determine the in-plane optical anisotropy, the optical parameters including RI and absorption coefficient of the sample along different in-plane directions should be precisely measured. In this work, we propose an incident angle scanning method to retrieve not only the complex RIs but also the thickness of thin film samples. Since a pair of 2D scanning galvanometers is involved in the experimental setup of ASE-SPRHM (Fig. 1d and Supplementary Information S.2), the incident angle of excitation light *θ* can be freely adjusted apart from the in-plane angle *ϕ*.

**Fig. 2:**
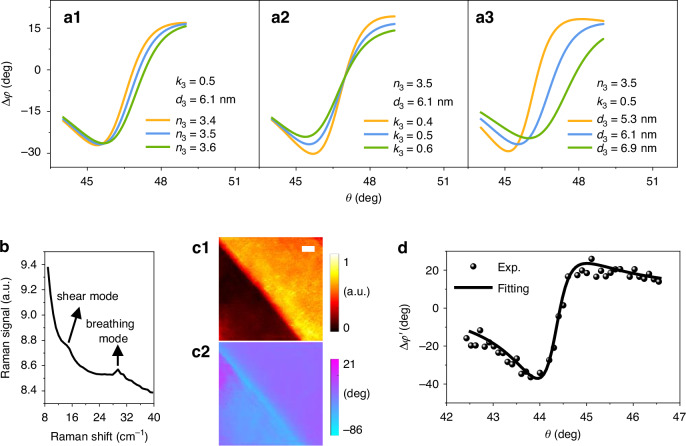
Principle and experiment result of retrieving complex RIs and thickness of ReS_2_ sample. **a** Reflection phase shift difference Δ*φ* of film sample versus incident angle of excitation light *θ* for different *n*_3_, *k*_3_ and *d*_3_ of the sample calculated by using Fresnel formulae. Δ*φ* is obtained by subtracting the reflection phase shifts when the dielectric above film sample is water and air. **b** Low-frequency Raman spectra of the measured sample. **c1**, **c2** SPR intensity and phase images of the sample at *ϕ=*0 and *θ=θ*_SPR_. **d** Measured reflection phase shift differences Δ*φ'* of the sample area versus incident angle *θ*. The dots represent experimental data and the curve is the fitting result. Scale bars in (**c**): 5 μm

According to the Fresnel formulae, the reflection coefficient *r* is determined by the complex RIs ${\tilde{n}}_{3}$ and thickness *d*_3_ of 3^rd^ dielectric layer above the metallic film surface if the other parameters of Kretschmann configuration are fixed. The incident angle of excitation light *θ* is slightly adjusted nearby the SPR angle when the sample area is under SPR condition. Figures 2a1–a3 show the SPR curves of Δ*φ* versus incident angle *θ* for different real part *n*_3_, imaginary part *k*_3_ of complex RIs and thickness *d*_3_ of the film sample, respectively. It’s obvious that the varieties of *n*_3_, *k*_3_ and *d*_3_ have different effects on the theoretical curves of Δ*φ* - *θ*. Consequently, the dependence of Δ*φ* - *θ* curves on ${\tilde{n}}_{3}$ and *d*_3_ can be used to quantitatively retrieve the complex RIs and thickness of film sample. Specifically, as the incident angle *θ* changes, the theoretical values of Δ*φ* are calculated from the Fresnel formulae and the corresponding experimental data Δ*φ'* are measured by SPRHM. Both of the ${\tilde{n}}_{3}$ and *d*_3_ of film sample will be retrieved by fitting the experimental data with the theoretical values using the least-square fitting method

1 $$\mathop{\min }\limits_{{\tilde{n}}_{3},{d}_{3}}\mathop{\sum }\limits_{m=1}^{m=M}{[\Delta {{\varphi }^{{\prime} }}_{m}({\theta }_{m})-\Delta {\varphi }_{m}({\theta }_{m},{\tilde{n}}_{3},{d}_{3})]}^{2}$$

where *M* represents the total number of incident angles in experiments and *m* denotes the *m*^th^ incident angle.

To demontrate the effectiveness of this method, a few-layer ReS_2_ sample was prepared on the metallic film surface and low-frequency Raman spectroscopy was used to characterize the sample layer in advance. The Raman spectra in Fig. 2b shows an obvious breathing mode signal at 29.46 cm^−1^ and a weak shear mode signal near 15 cm^−1^. The two modes indicate that the sample has two layers^66^. A series of SPR intensity and phase images of the two-layer (2 L) ReS_2_ sample was obtained by using SPRHM when the in-plane angle of excitation light *ϕ=*0 and the incident angle *θ* ranged from 42.4^o^ to 46.5^o^. Figures 2c1 and c2 show the results when *θ* equals to the SPR angle. The average values of reflection phase shift difference Δ*φ'* of one randomly selected sample area were calculated from the SPR phase images and plotted with *θ* by dots in Fig. 2d. By using the least-square fitting method discussed above, the sample complex RIs ${\tilde{n}}_{3}$ and thickness *d*_3_ were retrieved by the fitting curve in Fig. 2d. In order to quantify the measurement errors, we randomly selected other three sample areas and used the data to perform the fitting procedures (see Supplementary Information S.4 for details). Standard deviations of the total four groups of retrieved real part *n*_3_, imaginary part *k*_3_ of complex RIs and thickness *d*_3_ are calculated as 0.15, 0.03 and 0.04 nm, respectively. Thus, the corresponding measurement results should have two significant digits after the decimal point for *n*_3_, *k*_3_ and *d*_3_. Averages of the four groups of results are calculated as *n*_3_ = 4.56, *k*_3_ = 0.54, *d*_3_ = 1.55 nm and serve as the final measurement results of complex RIs and thickness of the film sample.

Since 1 L ReS_2_ has a thickness of about 0.7 nm^20^ or 0.8 nm^37^ which was measured on the smooth SiO_2_/Si substrate by using AFM. Thus, the 2 L ReS_2_ sample used in this experiment has a thickness of about 1.5 nm in theory, which matches well with our result of 1.55 nm. As for the complex RIs ${\tilde{n}}_{3}$, to the best of our knowledge, only one group proposed to use an empirical equation to calculate the ${\tilde{n}}_{3}$ of ReS_2_ flakes by fitting the data of angle-resolved polarized optical contrast spectroscopy^48^. Mono- to three-layer ReS_2_ samples were measured in that work and the complex RIs of 2 L sample along and perpendicular to the Re-Re chain direction were 3.88 + 0.50i and 4.25 + 0.88i at the wavelength of 632.8 nm, respectively. The imaginary part of our result *k*_3_ = 0.54 falls in the range of 0.50–0.88. While the real part *n*_3_ = 4.56 has a slight discrepancy with their range of 3.88–4.25 which may originate from the fabrication method of samples and the measurement method^55^.

It is noted that during the retrieval process of ${\tilde{n}}_{3}$ and *d*_3_, parameters of Kretschmann configuration are regarded as constants. To investigate the sensitivity of the fitting to potential variations in constant parameters, such as the gold film’s thickness and permittivity, we artificially added several groups of variations in gold film’s thickness (δ*d*_2_) and permittivity (δ*ε*_2_*'*, δ*ε*_2_*''*) and performed the fitting procedures to retrieve the corresponding complex RIs (*n*_3_, *k*_3_) and thickness (*d*_3_) for the 2 L sample. The induced errors δ*n*_3_, δ*k*_3_ and δ*d*_3_ are plotted with δ*d*_2_, δ*ε*_2_*'* and δ*ε*_2_*''* in Supplementary Information S.5. The results indicate that the retrieved real part of RIs of the sample is the most sensitive to the variation in gold film’s thickness.

The influence of experimental limits on the retrieval accuracy of ${\tilde{n}}_{3}$ and *d*_3_ is mainly coming from the measurement error of reflection phase shift difference Δ*φ'*. We characterized the measurement error of Δ*φ'* as 11.8 mrad by using the experimental setup. Gaussian phase noises with a level of 11.8 mrad were artificially added to the original experimental data Δ*φ'* in Fig. 2d and the corresponding ${\tilde{n}}_{3}$ and *d*_3_ were retrieved again. The result suggests that the measurement error of Δ*φ'* induces retrieval errors on real part *n*_3_, imaginary part *k*_3_ of complex RI and thickness *d*_3_ of 0.04, 0.01, 0.02 nm, respectively. (see Supplementary Information S.6 for details).

### Quantitative determination of in-plane optical anisotropy of ReS_2_ samples

**Fig. 3:**
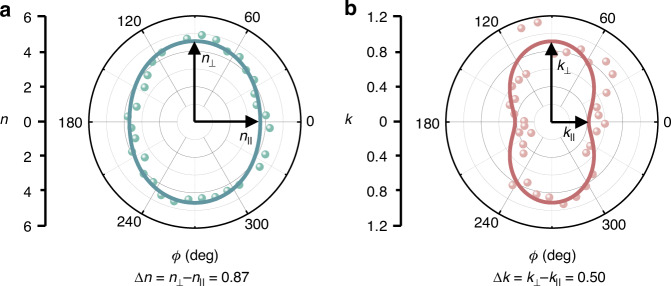
Measurement results of complex RIs of 2 L ReS_2_ sample at the in-plane angles around a circle. The in-plane optical anisotropy can be quantitatively determined by calculating the birefringence Δ*n* and dichroism Δ*k*. **a** Real part *n* and **b** imaginary part *k* of complex RIs versus in-plane angle *ϕ*

By changing the in-plane angle *ϕ* around a circle at an interval of 10 degrees in sequence, the 2 L ReS_2_ sample was measured by using the experimental setup of ASE-SPRHM and above retrieval method. Figure 3a, b show the results of real part *n* and imaginary part *k* versus in-plane angle *ϕ* in polar plots, respectively. The dots and closed lines represent the experiment and fitting results, respectively. The experimental data were the average values of one randomly selected sample area which is consistent with that in Fig. 2d. The fitting method was the same with that of Δ*φ'* data in Fig. 1e. As expected, the closed lines in Fig. 3 also show elliptical shapes. Here, to display the in-plane optical anisotropy more clearly, the long axes of elliptical shape plots are rotated manually along the vertical directions. The complex RIs along the long and short axes are denoted as ${n}_{\perp },{k}_{\perp }$ and ${n}_{||}$,${k}_{||}$, respectively. Considering the crystallographic structure of ReS_2_, ${n}_{\perp },{k}_{\perp }$ and ${n}_{||}$,${k}_{||}$ represent the complex RIs along and perpendicular to the Re-Re chain directions, respectively^48^. The birefringence and dichroism are thus calculated as ${\Delta} n={n}_{\perp }-{n}_{||}=0.87$ and ${\Delta} k={k}_{\perp }-{k}_{||}=0.50$ which reflect the magnitude of in-plane optical anisotropy.

**Fig. 4:**
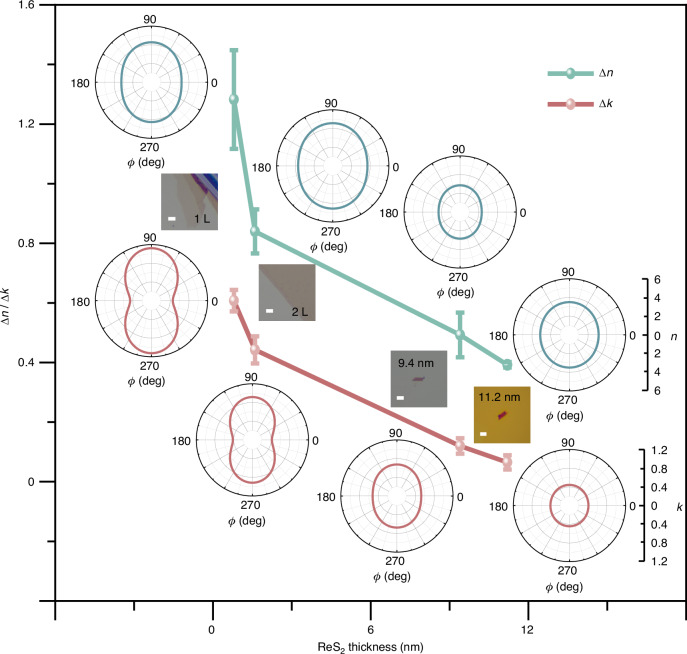
In-plane optical anisotropy of ReS_2_ samples with various thicknesses. The inserts are the optical microscopic images of samples and the polar plots aside each image are the real part *n* and imaginary part *k* versus in-plane angle *ϕ*, respectively. Scale bars: 5 μm

Layer-dependent properties of ReS_2_ 2D material, such as angle-resolved Raman response^20^, photothermal signal^36^, SHG signal^37^, *etc*., have drawn great research interests in recent years. Here, we investigate the layer-dependent in-plane optical anisotropy of ReS_2_ samples for the first time. Firstly, monolayer (1 L) ReS_2_ sample was prepared and measured. The optical microscopic image is shown in the insert of Fig. 4 which is denoted by “1 L”. Two polar plots aside the image are the measurement results of real part *n* and imaginary part *k* versus in-plane angle *ϕ*, respectively. Compared to the 2 L sample, the 1 L one has a relatively larger in-plane optical anisotropy with Δ*n* = 1.28 and Δ*k* = 0.61. The closed lines in two polar plots are the fitting results and the original experiment results can be seen in Supplementary Information S.7. Furthermore, two multilayer samples with thickness of 9.4 nm and 11.2 nm were prepared and measured (see the thickness characterization results by AFM in Supplementary Information S.8). Again, the optical microscopic images and polar plots of complex RIs versus in-plane angle *ϕ* are displayed in the inserts of Fig. 4. The raw experiment results of complex RIs of the 2 L and two multilayer samples are shown in Supplementary Information S.9. To quantify the error of fitting process, we randomly selected different sample areas to retrieve the complex RIs for the above four samples. The corresponding anisotropy parameters are calculated and the error bars are added in Fig. 4. It can be observed that as the sample thickness increases, the elliptical shapes of Δ*n* and Δ*k* in polar plots become nearly circular. That means the magnitude of in-plane optical anisotropy of ReS_2_ decreases with the increase of sample thickness.

It’s intuitive that multi-layer ReS_2_ should have the same high in-plane optical anisotropy as that of the monolayer one if the samples are exfoliated from the bulk crystal ReS_2_. However, a recent publication reports that bulk and few-layer ReS_2_ samples have distinct optical property, suggesting that bulk ReS_2_ cannot be assumed as decoupled monolayers^67^. The reason lies in that two polymorphic phases AA and AB co-exist in a single ReS_2_ crystal. In addition, another recent publication also reports that ReS_2_ crystals can stack along both the *c*-axis and *a*-axis, resulting in various stacking orders. Multiple stacking orders in few-layer ReS_2_ are coexisting^68^. From this point of view, different polymorphic phases can coexist in the layered ReS_2_ samples which were used in this work. The more layers the sample has, the more disordered stack becomes and the smaller the in-plane optical anisotropy is. Furthermore, a relative transition-metal dichalcogenide of ReS_2_, i.e., MoS_2_ has been reported to have highly anisotropic response as the thickness is reduced to monolayer^69^. Our finding of decreased anisotropy with the increase of ReS_2_ thickness will give a guidance to choose appropriate layer number of 2D materials in engineering nanodevices related to in-plane optical anisotropy.

## Discussion

The thicknesses of two multilayer samples were measured by AFM in advance and the values were put into the retrieval procedure of complex RIs. The reason lies in that the retrieval method is based on the least-square fitting approach, fewer unknown variables give more precise fitting results under the same convergence criteria. The thickness limitation of samples by using our method is determined by the penetration depth of SPW which is usually 200 to 300 nm. If the sample thickness is beyond the penetration depth of SPW, our method can detect the sample information in near field region. At this time, the four-layer SPR model used for above thin film samples will become three-layer SPR model^53,60^. However, regardless of which model is used, it is important to emphasize that their working principles require to excite SPP. Specifically, the RI of thicker samples can be detected by using three-layer SPR model with the condition that the RI of sample is smaller than that of the glass substrate. While, as for four-layer model, SPP can be excited even though RI of thin film sample is larger than that of the glass substrate, like the 2D materials investigated in this work. The reason lies in that the film sample is very thin and the evanescent wave can transmit through the film sample to excite SPP (see Supplementary Information S.10 for details).

Since the inherent transverse propagation of SPW occurs along the metallic surface, “tail” patterns exist in SPR images. This results in the spatial resolution of SPRHM under single direction of excitation being only tens of micrometers. But if SPR images under each direction of excitation are obtained by ASE method in ASE-SPRHM, followed by the image fusion procedure, the spatial resolution of SPR images can reach the diffraction limit of coherent imaging system^62^. In this work, the role of ASE is to obtain the individual SPR images under each direction of excitation, which were used to present the sample properties at different in-plane directions by retrieving the complex RIs and calculating the in-plane optical anisotropy. Thus, there is no image fusion procedure to obtain high-resolution SPR images and the spatial resolution of present usage is tens of micrometers.

As for the temporal resolution, for each excitation direction, an incident angle scanning range of 4.1 degrees with an interval of 0.1 degree, i.e., 41 incident angles are required to retrieve the complex RI. To include all of the azimuthal angles covering a circle, 36 rounds of incident angle scanning procedures are conducted. Consequently, a total of 2952 frames of holograms (41*36*2) are recorded, where number 2 indicates that corresponding reference holograms are also recorded. From the testing data of our setup, the fastest time is 70 ms to record one frame of hologram. In a word, 3.44 min are needed to obtain the final result of in-plane optical anisotropy for one sample. From this point of view, the current experimental setup is only applicable to samples with slowly changing properties and not applicable to dynamic scenarios.

In conclusion, we have demonstrated a near-field method, i.e., ASE-SPRHM, to quantitatively determine the in-plane optical anisotropy of low-dimensional materials. This method generates near-field SPWs oscillating along various in-plane directions by azimuthal scanning excitation of SPR. The utilization of near-field light-matter interactions between SPWs and low-dimensional material samples results in highly sensitive SPR detection signal. Using the dependence of SPR reflection phase on the near-field sample’s physical parameters, the complex RIs of low-dimensional material sample along all of the in-plane directions can be quantitatively retrieved with a designed incident angle scanning method of SPRHM. Compared with the traditional methods which use light-matter interactions between far-field light and material samples and rely on long interaction distance to acquire high signal-to-noise ratio, our method is applicable to ultrathin films down to atomic layer. A representative 2D material, i.e., layered ReS_2_ with various thicknesses have been used to verify the validity of the proposed method, and it’s found that the magnitude of in-plane optical anisotropy decreases with the increase of sample thickness. Since this is the first report, to the best of our knowledge, about the quantitative experiment study of layer-dependent in-plane optical anisotropy, further interests may be drawn from experts in the fields of optics, materials and nanophotonics. Our work provides a quantitative and accurate determination method for in-plane optical anisotropy of not only low-dimensional materials but also other thin films. This method will play an important role in finding new anisotropic low-dimensional materials and give a guidance in engineering new polarized nanodevices. Furthermore, the proposed method possesses the advantage of wide-field imaging modality which has the potential to investigate the distribution of in-plane optical anisotropy for more complex sample systems.

## Materials and methods

### Materials

The SPR configuration was made up with coverslip coated with metallic film and sample. A 170 μm thick coverslip (BK7 glass) was cleaned with acetone, ethanol, and deionized water successively in ultrasonic cleaners. After that, the coverslip was deposited with 1 nm Cr layer as the protecting layer and 45 nm gold as the SPR excitation layer using magnetron sputtering at speed of 0.01 nm/s and 0.03 nm/s, respectively. The ReS_2_ samples were prepared by mechanical exfoliation.

### Methods

The thickness of 1 L and 2 L ReS_2_ samples was characterized by measuring the low-frequency Raman spectroscopy with the instrument of WITec Alpha 300 R, the magnification of objective is 100x and the excitation wavelength is 532 nm. The thicknesses of muti-layer samples were characterized by AFM.

## Supplementary information

Supplementary Information

## Data Availability

The data that support the findings of this study are available from the corresponding author upon reasonable request.
