# Supplementary material for: Quantitative determination of in-plane optical anisotropy by surface plasmon resonance holographic microscopy
Source: Light Sci Appl. 2026 Mar 6;15:152. doi: 10.1038/s41377-026-02207-7 (PMC12966428; doi:10.1038/s41377-026-02207-7)
Supplement: Supplementary file 1 — Supplementary Information [file 41377_2026_2207_MOESM1_ESM.docx]

Supplementary Information

**Quantitative Determination of In-plane Optical Anisotropy by Surface Plasmon Resonance Holographic Microscopy**

Jiwei Zhang^1^, Wenrui Li^1^, Jiahao Li^1^, Yujie Zhang^1^, Xiaoqing Chen^2^, Xiangyuan Luo^1^, Siqing Dai^1^*, Xuetao Gan^1^*, and Jianlin Zhao^1^*

^1^*Key Laboratory of Light Field Manipulation and Information Acquisition, Ministry of Industry and Information Technology, and Shaanxi Key Laboratory of Optical Information Technology, School of Physical Science and Technology, Northwestern Polytechnical University, Xi’an 710129, China*

^2^*School of Artificial Intelligence, Optics and Electronics (iOPEN), Northwestern Polytechnical University, Xi’an 710129, China*

*E-mail: siqing.dai@nwpu.edu.cn*

*E-mail: xuetaogan@nwpu.edu.cn*

*E-mail: [jlzhao@nwpu.edu.cn](mailto:jlzhao@nwpu.edu.cn)*

S.1 Fresnel formulae

As for the four-layer SPR model used in this work, the reflection coefficient *r*_1,_*_N_* (*N*=4) of Kretschmann configuration is described by the Fresnel formulae

*$\begin{matrix} r_{i,N}\left(\theta\right)=\frac{r_{i,i+1}\left(\theta\right)+r_{i+1,N}\left(\theta\right)\exp\left[2\text{j}d_{i+1}k_{zi+1}\left(\theta\right)\right]}{1+r_{i,i+1}\left(\theta\right)r_{i+1,N}\left(\theta\right)\exp\left[2\text{j}d_{i+1}k_{zi+1}\left(\theta\right)\right]} \\ \text{ }\left(\text{j=}\sqrt{-1},i=1,2,...N-2\right) \end{matrix}$* (S1a)

*$r_{i,i+1}\left(\theta\right)=\frac{\xi_{i+1}\left(\theta\right)-\xi_{i}\left(\theta\right)}{\xi_{i+1}\left(\theta\right)+\xi_{i}\left(\theta\right)},\left(i=1,2,...N-1\right)$* (S1b)

*$\xi_{i}\left(\theta\right)=\varepsilon_{i}/k_{zi}\left(\theta\right),\left(i=1,2,...N\right)$* (S1c)

*$k_{zi}\left(\theta\right)=2\text{π}\frac{\sqrt{\varepsilon_{i}-\varepsilon_{1}\sin^{2}\left(\theta\right)}}{\lambda},\left(i=1,2,...N\right)$* (S1d)

where *i* represents the *i*th dielectric, *r_i_*_,_*_i_*_+1_ the reflection coefficient between two adjacent dielectrics, and *k_zi_* the wavenumber of transmission light along the *z* direction in the *i*th dielectric. The reflectivity and reflection phase shift are

*$R\left(\theta\right)=\left|r_{1,N}\left(\theta\right)\right|^{2}$* (S2)

** (S3)

S.2 Experimental setup of ASE-SPRHM

The azimuthal scanning excitation SPR holographic microscopy (ASE-SPRHM) was previously proposed to improve the spatial resolution of SPR images^62^. In this work, the experimental setup of ASE-SPRHM has been updated which is shown in **Fig. S1**.

**Fig. S1** Experimental setup of ASE-SPRHM

The experimental setup uses wavevector and polarization correlated light fields to excite SPR and common-path hologram recording structure to acquire SPR intensity and phase images. A pair of scanning galvanometers with orthogonal rotating axes are used to adjust the incident angle *θ* and in-plane angle *φ* of excitation light beam. A vortex half-wave plate (HWP) is used to ensure that the incident beam has both *s*- and *p*-polarization components with equal amplitude for any in-plane angles. The *p*-polarization component excites SPR, and SPW propagating in arbitrary direction on the gold film can be generated. Two identical mirrors 2 and 3 with orthogonal incident planes are used to direct the incident beam into the objective. This ensures that the excitation light beam on the Kretschmann configuration has the right polarization state at all of the in-plane angles.

The beam reflected by Kretschmann configuration carries the sample information and the sample is imaged on the CCD target by lens 4. For any in-plane angle, the excitation beam and reflected one pass through the vortex HWP symmetrically and the corresponding fast axes are perpendicular to each other. Thus, the polarization direction of reflected beam will be rotated to the same as that of incident beam. A Wollaston prism splits the vertical and horizontal, *i.e.*, *s*- and *p*-polarization components of reflected beam with a small angle, of which the *s*-polarization component cannot excite SPR and thus plays as the reference beam. The overlapped area of two beams forms the off-axis hologram after passing through a polarizer.

S.3 Measurement results of in-plane isotropic graphene

**Fig. S2** Measurement results of (a) SPR intensity image, (b) SPR phase image and (c) reflection phase shift difference Δ*ϕ'* of the in-plane isotropic graphene sample versus in-plane angle *φ* when the 4^th^ dielectric above sample is water and air.

S.4 Fitting results of complex RIs and thickness of 2L ReS_2_ sample

**Fig. S3** Experiment results of reflection phase shift differences Δ*ϕ'* versus incident angle *θ* at four randomly selected areas of 2L ReS_2_ sample. The dots represent experimental data and the curves are fitting results. The retrieved complex RIs and thickness of sample are shown in the insets of each figure. The values of R^2^ in each figure are coefficients of determination which indicate the goodness of fit. The closer R^2^ is to 1, the better the fitting.

S.5 Sensitivity of fitting to potential variations in gold film’s thickness and permittivity

**Fig. S4** Retrieval errors of complex RIs (δ*n*_3_, δ*k*_3_) and thickness (δ*d*_3_) of thin film sample caused by potential variations in gold film’s thickness (δ*d*_2_) and permittivity (δ*ε*_2_*'*, δ*ε*_2_*''*).

S.6 Influence of experimental limits on the measurement accuracy of $\tilde{n}_{3}$ and *d*_3_

To characterize the measurement error of Δ*ϕ'*, 81 frames of holograms were recorded successively without the sample at a speed of 15 fps. Then phase difference distributions of the latter 80 frames were calculated by subtracting the reconstructed phase distribution of the first hologram from that of the latter 80 holograms. After that, a same random square area containing 2500 pixels was selected in every phase difference distribution, and the phase difference standard deviation of the whole 80 frames for each pixel was calculated. **Figure S5** depicts the histogram of these standard deviations, and the blue fitting curve turns out to be nearly a Gaussian type. The mathematical expectation of the deviations indicates that phase fluctuation of the setup is 11.8 mrad which is usually regarded as the measurement error of Δ*ϕ'*. The reason of recording 81 frames of holograms at a speed of 15 fps lies in the raw data recording process for once measurement. Specifically, an incident angle scanning range of 4.1 degrees with an interval of 0.1 degree, (*i.e.*, a total of 41 incident angles) is required to retrieve the complex RIs and thickness. Consequently, a total of 82 holograms (41*2) are recorded, where number 2 indicates that corresponding reference holograms are also recorded. From the testing data of our setup, the fastest time is 70 ms to record one hologram when the 2D scanning galvanometers are working. In a word, 82 holograms in 5.74 seconds are needed to obtain the result of the complex RIs and thickness for one sample.

**Fig. S5** Measurement accuracy of Δ*ϕ'*

Taking the 2L ReS_2_ as an example, we artificially add Gaussian phase noises with a level of 11.8 mrad to the original experimental data Δ*ϕ'* in Fig. 2d and retrieve the corresponding complex RIs and thickness. The original fitting result and the one added noise are shown in **Fig. S6**. The result suggests that the measurement error of Δ*ϕ'* induces retrieval errors on real part *n*_3_, imaginary part *k*_3_ of complex RI and thickness *d*_3_ of 0.04, 0.01, 0.02 nm, respectively.

**Fig. S6** (a) Original experimental data of reflection phase shift differences Δ*ϕ'* versus incident angle *θ* of 2L ReS_2_ sample in Fig. 2d. (b) Results that Gaussian phase noises with a level of 11.8 mrad are added in (a). The dots represent experimental data and the curves are fitting results.

S.7 Measurement results of complex RIs of 1L ReS_2_ sample

**Fig. S7** Measurement results of real part *n* and imaginary part *k* of 1L ReS_2_ versus in-plane angle *φ*, respectively. (a-d) Results retrieved by using the experimental data from four randomly selected areas of the sample. Since the prepared 1L sample is located aside the sample with other thicknesses, only 18 in-plane angles of SPR excitation can be realized.

Detailed explanation about only 18 in-plane angles measured for the 1L ReS₂ sample: The reason lies in that a “tailing” pattern accompanies the sample along the propagation direction of SPP under the single excitation^62^. If there are undesired samples located besides the sample to be measured, the “tailing” patterns caused by the undesired samples will appear in the region of interest and induce fake results. The 1L ReS₂ sample prepared by mechanical exfoliation in this work is accompanied by samples with thicker thickness, as shown in the insert of Fig. 4. In this case, half number of azimuthal angles, *i.e.*, 18 angles covering a half circle were used.

This reduced angular sampling does not affect the accuracy of fitting procedure. The reason is that the fitting accuracy is affected by the sampling interval, not by the sampling range. In the whole manuscript, the sampling interval of azimuthal angle is kept as 10 degrees. Actually, to quantitatively characterize the in-plane optical anisotropy, two complex RIs of the sample along and perpendicular to the Re-Re chain are sufficient. This means that if one can identify the direction of Re-Re chain in advance, angular sampling covering a quarter of circle is sufficient. In our work, priori knowledge about the direction of Re-Re chain is lacking. Thus, 18 azimuthal angles covering a half circle which include the directions along and perpendicular to the Re-Re chain are used. Regarding the angles covering the other half circle, the obtained data are symmetric to those obtained at the first half circle. In a word, 18 azimuthal angles covering a half circle are sufficient to retrieve the in-plane optical anisotropy with high accuracy.

S.8 Thickness characterization results of two multi-layer samples by AFM

**Fig. S8** (a) Optical microscopic image and (b) thickness characterization result of 9.4 nm thick ReS_2_ sample by AFM. (c) and (d) The results of 11.2 nm thick ReS_2_ sample. Scale bars: 5 μm.

S.9 Measurement results of complex RIs of 2L, 9.4 nm and 11.2 nm ReS_2_ samples

**Fig. S9** Measurement results of real part *n* and imaginary part *k* of 2L ReS_2_ versus in-plane angle *φ* for four sample areas 1-4. (a, c, e, g) Real part *n* and (b, d, f, h) imaginary part *k* of complex RIs versus in-plane angle *φ*, respectively.

**Fig. S10** Measurement results of real part *n* and imaginary part *k* of 9.4 nm thick ReS_2_ versus in-plane angle *φ* for four sample areas 1-4. (a, c, e, g) Real part *n* and (b, d, f, h) imaginary part *k* of complex RIs versus in-plane angle *φ*, respectively.

**Fig. S11** Measurement results of real part *n* and imaginary part *k* of 11.2 nm thick ReS_2_ versus in-plane angle *φ* for four sample areas 1-4. (a, c, e, g) Real part *n* and (b, d, f, h) imaginary part *k* of complex RIs versus in-plane angle *φ*, respectively.

S.10 Application scenarios for three- and four-layer SPR models

The difference between three- and four-layer SPR model is dependent on the thickness of sample to be measured. If the thickness of sample above the gold film is larger than the penetration depth of SPP, the SPR configuration is a three-layer model, *i.e.*, “glass-gold film-thick sample” model (**Fig. S12**(a)). Otherwise, it is a four-layer model of “glass-gold film-thin film sample-thick dielectric” (**Fig. 12**(b)) which is used in the present work. As for the three-layer SPR model, only the RI of thick sample could modulate the SPR condition and be detected sensitively by using the reflection intensity or phase shift signals. While, in the four-layer SPR model, both the thickness and RI of thin film sample could modulate the SPR condition and be detected. Regardless of which model is considered, it is important to emphasize that their working principles require to excite SPP. As for the three-layer model, SPP can be excited only when the RI of thick sample is smaller than that of the glass substrate because of the wave vector matching condition *$n_{1}\sin\theta=\sqrt{\frac{\varepsilon_{2}\varepsilon_{3}}{\varepsilon_{2}+\varepsilon_{3}}}$*, where *n*_1_ is the RI of glass substrate, *ε*_2_ and *ε*_3_ are the permittivity of gold film and thick sample, respectively, *θ* is the incident angle of excitation light. While, as for four-layer model, SPP can be excited even though RI of thin film sample is larger than that of the glass substrate, like the 2D materials investigated in the present work. The reason lies in that the film sample is very thin and the evanescent wave is able to transmit through the film sample to excite SPP.

In a word, the RI of thicker samples can be detected by using three-layer SPR model with the condition that the RI of sample is smaller than that of the glass substrate. 2D materials which usually have large RIs cannot be applied in the three-layer SPR model.

**Fig. S12** (a) Three- and (b) four-layer SPR models
